# Risk factors for hospital admission in the 28 days following a community-acquired pneumonia diagnosis in older adults, and their contribution to increasing hospitalisation rates over time: a cohort study

**DOI:** 10.1136/bmjopen-2015-008737

**Published:** 2015-12-01

**Authors:** Elizabeth R C Millett, Bianca L De Stavola, Jennifer K Quint, Liam Smeeth, Sara L Thomas

**Affiliations:** Faculty of Epidemiology and Population Health, London School of Hygiene and Tropical Medicine, London, UK

**Keywords:** EPIDEMIOLOGY, PUBLIC HEALTH

## Abstract

**Objectives:**

To determine factors associated with hospitalisation after community-acquired pneumonia (CAP) among older adults in England, and to investigate how these factors have contributed to increasing hospitalisations over time.

**Design:**

Cohort study.

**Setting:**

Primary and secondary care in England.

**Population:**

39 211 individuals from the Clinical Practice Research Datalink, who were eligible for linkage to Hospital Episode Statistics and mortality data, were aged ≥65 and had at least 1 CAP episode between April 1998 and March 2011.

**Main outcome measures:**

The association between hospitalisation within 28 days of CAP diagnosis (a ‘post-CAP’ hospitalisation) and a wide range of comorbidities, frailty factors, medications and vaccinations. We examined the role of these factors in post-CAP hospitalisation trends. We also looked at trends in post-CAP mortality and length of hospitalisation over the study period.

**Results:**

14 comorbidities, 5 frailty factors and 4 medications/vaccinations were associated with hospitalisation (of 18, 12 and 7 considered, respectively). Factors such as chronic lung disease, severe renal disease and diabetes were associated with increased likelihood of hospitalisation, whereas factors such as recent influenza vaccination and a recent antibiotic prescription decreased the odds of hospitalisation. Despite adjusting for these and other factors, the average predicted probability of hospitalisation after CAP rose markedly from 57% (1998–2000) to 86% (2009–2010). Duration of hospitalisation and 28-day mortality decreased over the study period.

**Conclusions:**

The risk factors we describe enable identification of patients at increased likelihood of post-CAP hospitalisation and thus in need of proactive case management. Our analyses also provide evidence that while comorbidities and frailty factors contributed to increasing post-CAP hospitalisations in recent years, the trend appears to be largely driven by changes in service provision and patient behaviour.

**Strengths and limitations of this study**

- Our novel use of large linked primary and secondary care data sets provided enriched patient medical and therapeutic histories, and allowed detailed identification of the determinants of hospitalisation after community-acquired pneumonia (CAP).
- The very large sample size, with more than 43 000 CAP episodes, enabled assessment of multiple potential risk factors for hospitalisation with precise estimation of relative risk.
- Using linked data also allowed us to distinguish trends in the tendency to hospitalise patients with CAP (the focus of this paper) from trends in CAP hospitalisations due simply to increasing CAP incidence.
- Our analyses suggested that frailty factors were suboptimally recorded by general practitioners, preventing full assessment of these factors and highlighting the need for better capture of frailty by practices.
- For similar reasons, analyses on the effect of smoking were performed on a subset of the data, due to previously described incomplete recording of smoking status pre-2004.

## Introduction

Hospitalisations for ambulatory care sensitive conditions (ACSC, conditions which could be treated outside of hospital) have increased considerably over the past decade.^1^ ^2^ Pneumonia is one such condition, with >56 000 more pneumonia admissions in 2010/2011 compared with 2001/2002.^2^ Most of this increased burden is found among patients aged 65 years and older who accounted for 70% of pneumonia admissions in 2012/2013.^3^ In England, the recently introduced Unplanned Admissions Enhanced Service highlights the importance of proactive case management in primary care of at-risk patients, many of whom are expected to be older, to reduce ACSC hospitalisations.^4^ ^5^

To date, risk factors for hospitalisation for community-acquired pneumonia (CAP) have not been quantified. Existing analyses based on stand-alone hospitalisation data are unable to compare hospitalised patients with CAP to those who were treated in the community. They therefore cannot distinguish between factors which affect a patient's likelihood of hospitalisation after a CAP diagnosis from risk factors for developing CAP. Furthermore, hospitalisation data have incomplete information on patients’ medical histories, and contain little or no information on factors such as alcohol and smoking habits, frailty, or medications prescribed in the community. It is frequently hypothesised that changes in comorbidities and frailty factors have contributed to the increasing hospitalisation trends for older individuals with CAP in the UK. Use of a non-hospitalised comparison group would allow this hypothesis to be tested, and to distinguish an increasing tendency to hospitalise older patients with CAP from increasing incidence of CAP among older adults.^6^

In this study, we used large linked general practice, hospital admission and mortality data sets to assess a variety of potential risk factors (comorbidities, medications and other factors) for hospitalisation after CAP among older individuals in England. Use of the general practice data enabled more complete capture of patient histories than those derived from stand-alone hospital records. We also investigated the risk over time of hospitalisation after a CAP diagnosis. The choice of a study population who had been diagnosed with CAP allowed us to examine specifically trends in hospitalisation after CAP, independent of any trends in pneumonia incidence. We assessed to what extent the patient factors associated with hospitalisation explained these hospitalisation trends. The linked mortality data enabled further investigation into whether mortality rates in the 28 days after CAP had changed over the same period, including deaths occurring both in and outside hospital settings, as a marker of CAP severity.

## Materials and methods

### Data sources

The Clinical Practice Research Datalink (CPRD, formerly GPRD) is a large database of UK general practice records comprising a representative sample of around 8% of the UK population.^7^ ^8^ Anonymised data in CPRD include diagnoses (coded using Read codes), prescriptions, referrals, tests and patient demographics. Over 50% of CPRD patients living in England have their general practice records linked to Hospital Episode Statistics (HES) which includes all inpatient National Health Service (NHS) hospitalisations (coded using International Classification of Diseases (ICD)-10).^9^ Each HES hospitalisation consists of one or more episode denoting the time a patient is under the care of one consultant. The data were also linked to Office for National Statistics (ONS) central mortality data to obtain vital status and, if relevant, date of death.

### Study population

We included patients who were registered with a CPRD practice eligible for linkage to HES data, were aged 65 years or over between 1 April 1998 and 31 March 2011, and who had a CAP episode recorded during that period.

### Defining CAP episodes

The methods for defining CAP illness episodes have been described in detail elsewhere.^6^ In brief, lists of Read (CPRD) and ICD-10 (HES) codes for pneumonia and other lower respiratory tract infections (LRTIs) were agreed by three clinical epidemiologists. Pneumonia could be first diagnosed either in general practice or when presenting to hospital. In HES, in order to differentiate between illness present at hospital admission and subsequent hospital-acquired illness, only pneumonia coded as the reason for admission (ie, the primary code in the first episode of a hospitalisation) was included in the study. These HES pneumonia records were combined with CPRD pneumonia records to determine ‘illness episodes’ whereby records within 28 days of each other (or of an intermediate LRTI record) were deemed part of the same infection.^6^ The earliest pneumonia record in the ‘illness episode’ was the incident (diagnosis) date of pneumonia.

To be defined as community-acquired, the CAP incident date needed to be ≥14 days after any HES inpatient hospital discharge. All CAP episodes in eligible patients during the study period were included in the study.

### Defining hospitalisation after pneumonia

The outcome of interest was hospital admission (defined using HES) for any cause, on or up to 28 days after the CAP diagnosis date. Thus, a CAP diagnosed when a patient presented at hospital was automatically assigned as having the outcome; a CAP diagnosed in general practice had the outcome if the patient had a hospital admission in the next 28 days. We chose all-cause hospitalisation because we also wanted to capture hospitalisations for events which pneumonia could have precipitated in our older population, such as stroke, myocardial infarctions and falls, or worsening of underlying comorbidities such as chronic obstructive pulmonary disease or congestive heart failure.^10^

### Other factors

Age was categorised in 5-year groups from 65 to 89 years, and ≥90 years.

#### Time period

Year of hospitalisation used a financial year structure (1 April to 31 March) to ensure respiratory pathogens circulating throughout the winter were captured in the same year. Year was then grouped as 1998–2000, 2001–2003, 2004–2006, 2007–2008 and 2009–2010, to account for health service changes such as the introduction of payment-for-performance indicators in 2004 and 2009.^11^ ^12^

#### Comorbidities and frailty factors

Code lists for the 19 comorbidities in the Charlson Index and for additional cardiac, neurological and immune disorders that could affect pneumonia disease severity or a doctor's decision to hospitalise were devised by author SLT and at least one other clinical epidemiologist.

Prevalidated frailty scores such as the frailty phenotype or frailty index could not be utilised due to aspects of each not being recorded in the electronic health records used in this study (eg, grip strength and slow gait speed from the phenotype and sucking problems and poor muscle tone in neck from the index).^13^ ^14^ Instead, a wide variety of factors identified in the frailty index as associated with frailty, for which information was potentially available in the databases, were considered. Authors ERCM and SLT devised Read and ICD-10 code lists and used other recording fields within the data to capture health deficits within the previous year (eg, history of falls, inability to self-care) which were likely to be recorded in patients’ health records, as well as other factors that could increase the likelihood of hospitalisation (eg, living alone).

The presence of chronic conditions (such as diabetes or dementia) was determined using CPRD and HES records from any point up to and including the CAP incident date. For acute/potentially acute conditions (myocardial infarction, stroke, congestive heart failure, hemiplegia, falls, weight loss/undernutrition) which could have occurred as a result of the CAP, records from any point prior to but excluding the CAP incident date itself were used as evidence of a pre-existing condition.

Terminal illness was defined using Read and ICD-10 codes stating terminal illness, rather than specific conditions. In addition, primary care information on referrals to hospices was included.

#### Medications, vaccinations and health behaviours

Medications included oral steroids, inhaled steroids, immunosuppressive drugs, statins and antibiotics. We also considered influenza and pneumococcal vaccination status, and health behaviours such as smoking and excessive alcohol consumption.

As is common when using routinely collected health records, for all the factors aforementioned, the absence of a code for a condition was assumed to represent absence of the condition.

A full list of the factors considered, how they were categorised and timescales used to determine if they were present at CAP diagnosis, can be found in online supplementary file A.

### Main analyses

Some patients had more than one CAP event during the study period. It is highly likely that decisions around whether to hospitalise a patient after CAP were affected by a previous history of CAP. Furthermore, decisions to hospitalise may have been similar for patients within a general practice, for example, due to local area service provision. To account for this clustering at patient and practice level, we used multilevel logistic regression models for the binary hospitalisation outcome.^15^ The model had three levels: CAP episodes nested within patients who were nested within practices. The suitability of the three-level model was assessed by comparing it to simpler specifications (using likelihood ratio tests (lrt)) both before and after including explanatory factors in the model.

First, minimally adjusted ORs of hospitalisation following a CAP (adjusted for age, sex and year of CAP) were produced for each of the factors of interest. The size of these ORs and their 95% CIs were used to decide which variables to include in later models. These variables were grouped into (1) comorbidities; (2) frailty factors; and (3) medications, vaccinations and health behaviours. We added the three groups of variables sequentially to a model adjusted for age, sex and year of CAP, according to each group's hypothesised place on the causal pathway to hospitalisation. This enabled examination of the independent effect of each comorbidity (in the ‘comorbidity’ model), and how much of each comorbidity's effect was explained by resulting frailty and/or medications (in subsequent models). A possible interaction between age and sex on the odds of hospitalisation was investigated in the final (full) model, comparing full models with and without the interaction term using an lrt.

To investigate the extent to which patient risk factors for hospitalisation explained trends in the probability of post-CAP hospitalisations, ORs for hospitalisation for each temporal period relative to 2001–2003 were estimated, controlling for changes in comorbidities and other factors. Multilevel models produce cluster-specific ORs of hospitalisation (ie, effects measured within each cluster). When investigating the level of hospitalisation after CAP over time, results at a population level were deemed more useful. Thus, we used the predicted cluster-level odds of hospitalisation derived from the final multilevel model to calculate the population average of predicted percentages of CAPs hospitalised in each year group.^16^

### Further analyses

Records for smoking enable the recording of a negative response (non-smoker), and so levels of missing data were able to be established for this variable. Smoking status was more completely recorded over time, decreasing from 26% missing data in 1998 to <1% by 2010 (see online supplementary file B). Multiple imputation was not considered appropriate as data were unlikely to be missing at random, for example, with respect to comorbidity status. Analyses including smoking as a covariate were therefore restricted to a subset of the data, performing a complete case analysis of CAP episodes with a recorded smoking status from 2007 onwards (which included more than 97% of CAPs per year).

Trends in mortality in the 28 days after CAP were assessed using the linked mortality data and multilevel logistic regression modelling (as for hospitalisation). Odds of mortality over time were adjusted for age and sex, but not for comorbidities or other factors, to avoid overadjustment of CAP severity resulting from underlying health deficits.

Two other potential explanations for trends in hospitalisation were investigated. The length of hospital admission for hospitalised patients over time (a further potential proxy of severity of illness) was examined using HES data. The pathway of care for each CAP episode over time was assessed by examining method of admission data in HES, and whether there was a general practice consultation for CAP (or potential CAP) on the day of diagnosis. We widened the definition of CAP in general practice to any LRTI to allow for conservative coding by general practitioners (GPs) in the absence of radiographical confirmation of pneumonia.^17^

All analyses were performed using Stata MP V.11.2.

## Results

Of 917 859 potentially eligible patients, 39 211 had at least one recorded CAP and their 43 576 CAP illness episodes were included in the study. The median age at diagnosis was 81 years (lower-upper quartiles: 75–87 years) and 53% of CAPs were in females (table 1). Most patients (91%) experienced one CAP, 7% of patients had two episodes and 2% had 3–8 episodes. The reason for admission was coded with an ICD-10 Chapter X code (Diseases of the respiratory system) for 95% of admissions throughout the study period, with Chapter XVIII (Signs and symptoms not elsewhere classified) and Chapter IX codes (Circulatory disease) each contributing around 1% of admissions.

**Table 1:** Characteristics of the study population, factors of interest and hospitalisation within 28 days of CAP

	Hospitalised within 28 days n	Not hospitalised n	Total
All CAPs n (%)	33 321 (76.5)	10 255 (23.5)	43 576
Male n (%)	16 143 (79.5)	4151 (20.5)	20 294
Female n (%)	17 178 (73.8)	6104 (26.2)	23 282
Age (grouped) n (%)			
65–69	3469 (75.9)	1099 (24.1)	4568
70–74	4703 (78.8)	1262 (21.2)	5965
75–79	6039 (78.4)	1663 (21.6)	7702
80–84	7227 (79.5)	1865 (20.5)	9092
85–89	6666 (76.6)	2038 (23.4)	8704
90+	5217 (69.1)	2328 (30.9)	7545
Year of CAP (grouped) n (%)			
1998–2000	4008 (57.7)	2944 (42.3)	6952
2001–2003	6266 (69.9)	2701 (30.1)	8967
2004–2006	8269 (79.2)	2173 (20.8)	10 442
2007–2008	7039 (83.7)	1372 (16.3)	8411
2009–2010	7739 (87.9)	1065 (12.1)	8804
*Individual comorbidities n (%)*			
Ischaemic heart disease			
Pre-MI	7261 (81.5)	1644 (18.5)	8905
Post-MI	4914 (83.2)	994 (16.8)	5908
Congestive heart failure	8289 (79.6)	2124 (20.4)	10 413
Peripheral vascular disease	4661 (82.7)	976 (17.3)	5637
Dementia	4526 (66.8)	2248 (33.2)	6774
Chronic lung disease	14 571 (83.4)	2905 (16.6)	17 476
Connective tissue disease	3347 (81.9)	740 (18.1)	4087
Peptic ulcer	3343 (81.1)	778 (18.9)	4121
Liver disease			
Mild	241 (84.3)	45 (15.7)	286
Severe	165 (87.3)	24 (12.7)	189
Diabetes			
Diabetes	4678 (81.3)	1076 (18.7)	5754
With complications	1633 (86.6)	253 (13.4)	1886
Hemiplegia	1243 (76.4)	384 (23.6)	1627
Cancer			
Solid cancer	5208 (80)	1300 (20)	6508
Metastatic	1066 (83.9)	204 (16.1)	1270
Leukaemia/lymphoma	981 (85)	173 (15)	1154
Severe renal disease	7001 (88.6)	900 (11.4)	7901
Cerebrovascular disease	8338 (74.5)	2856 (25.5)	11 194
Neurological disease	2997 (73.1)	1103 (26.9)	4100
Disorders of the immune mechanism	243 (90)	27 (10)	270
Mild renal disease	401 (82.5)	85 (17.5)	486
Terminal illness	1190 (67.1)	584 (32.9)	1774
*Frailty factors n (%)*			
Recent carer	1418 (79.6)	364 (20.4)	1782
Living arrangements			
Not recorded	27 949 (77.6)	8070 (22.4)	36 019
Lives alone	1471 (81)	344 (19)	1815
Sheltered accommodation	477 (79)	127 (21)	604
Residential care	3424 (66.6)	1714 (33.4)	5138
Visual impairment	11 098 (78.8)	2984 (21.2)	14 082
Self-care	366 (79.7)	93 (20.3)	459
Anxious/depressed	2730 (76)	860 (24)	3590
Bedsore/ulcer	824 (59.7)	556 (40.3)	1380
Mobility issues	2072 (79)	552 (21)	2624
Tired	1957 (74.4)	672 (25.6)	2629
Low weight/poor nutrition	4460 (75.1)	1477 (24.9)	5937
Incontinence/catheter	3230 (71.7)	1274 (28.3)	4504
History of falling	4792 (76.4)	1484 (23.6)	6276
Excessive alcohol consumption			
Any excess alcohol code	1720 (80.3)	423 (19.7)	2143
*Medications n (%)*			
Immunosuppressants (other than steroids) in past 120 days	685 (85.3)	118 (14.7)	803
Inhaled corticosteroids			
None pre-CAP	22 414 (73.6)	8023 (26.4)	30 437
Within 60 days	6864 (84.7)	1239 (15.3)	8103
Within 61–180 days	1620 (81.6)	366 (18.4)	1986
Within 181–365 days	597 (81.4)	136 (18.6)	733
More than 365 days ago	1826 (78.8)	491 (21.2)	2317
Antibiotics			
None in previous 28 days	23 437 (77.2)	6926 (22.8)	30 363
In previous 1–7 days	5368 (76.9)	1610 (23.1)	6978
In previous 8–28 days	4516 (72.4)	1719 (27.6)	6235
Statins in previous 6 months	8829 (86.7)	1350 (13.3)	10 179
Oral steroids in previous 90 days	5242 (83)	1077 (17)	6319
Influenza vaccine receipt			
No vaccine pre-CAP	4940 (69.7)	2143 (30.3)	7083
14–365 days pre-CAP	20 554 (76.2)	6420 (23.8)	26 974
Last season	5990 (75.5)	1949 (24.5)	7939
2–5 years pre-CAP	1846 (71.7)	728 (28.3)	2574
>5 years pre-CAP	656 (76.4)	203 (23.6)	859
Pneumococcal vaccine			
No vaccine pre-CAP	13 126 (66.4)	6643 (33.6)	19 769
14–365 days pre-CAP	1872 (73.7)	669 (26.3)	2541
1–2 years pre-CAP	2095 (75.4)	682 (24.6)	2777
2–5 years pre-CAP	7260 (80.1)	1801 (19.9)	9061
>5 years pre-CAP	9633 (85.4)	1648 (14.6)	11 281

CAP, community-acquired pneumonia; MI, myocardial infarction.

### Risk factors for hospitalisation

After adjusting for age, sex and year, our study found little evidence that hemiplegia, mild renal disease, self-care problems, anxiety/depression, mobility issues, tiredness, history of falling or excessive alcohol consumption were risk factors for hospitalisation, and these factors were not included in subsequent analyses (see online supplementary file C).

**Figure 1:**
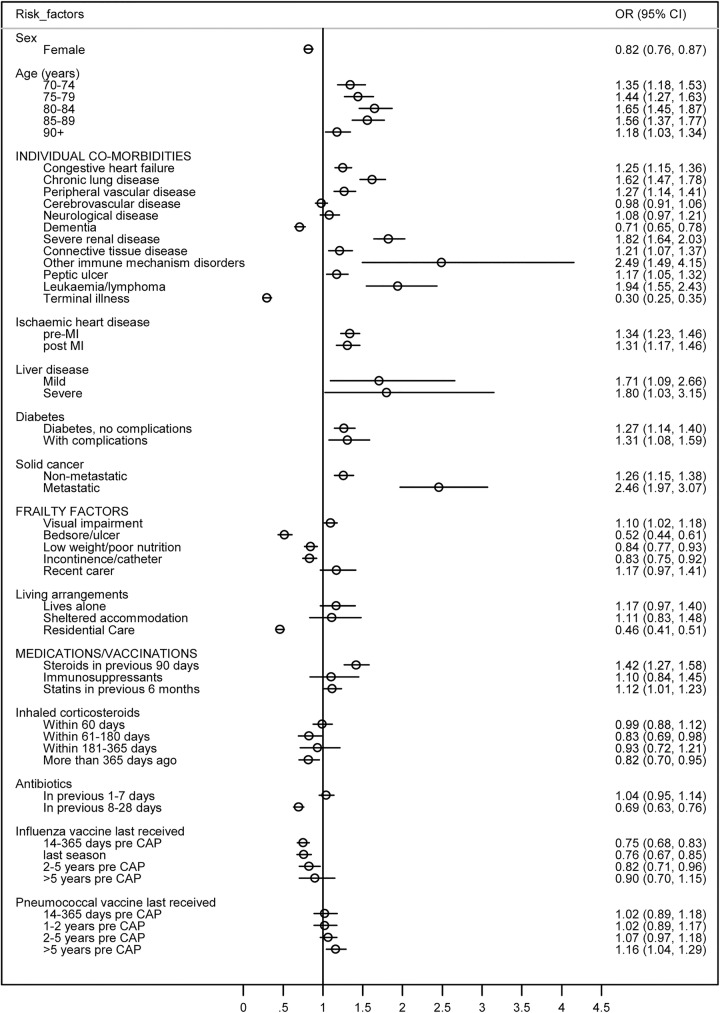
Mutually adjusted ORs (circles) with 95% CIs (lines) of hospitalisation in the 28 days after CAP for factors included in the final model*. The model also contained year of CAP diagnosis, but the results for year are not presented. *Baseline categories were age 65–69 years; condition or medication not present (for comorbidities, frailty factors, recent medications); unvaccinated/no record of vaccination (for influenza and pneumococcal vaccination) (CAP, community-acquired pneumonia; MI, myocardial infarction).

Results for the remaining 16 comorbidities, 6 frailty factors and 7 medications/vaccinations are given in figure 1 and in the online supplementary file C. The figure reports the final model in which ORs are mutually adjusted; the table reports minimally and then consecutively adjusted ORs in successive models.

#### Comorbidities

In the final model, 12 comorbidities were associated with increased odds of hospitalisation after CAP (figure 1). Of the comorbidities common among this cohort, chronic lung disease, ischaemic heart disease, congestive heart failure, severe renal disease and diabetes (with and without complications) were associated with a 25–82% increased odds of hospitalisation. The greatest odds of hospitalisation were found for less common conditions such as metastatic cancer and other disorders of the immune mechanism (adjusted ORs 2.46 and 2.49, respectively). Only terminal illness and dementia remained clearly associated with decreased odds of hospitalisation. Comparison with earlier models indicated that adjustment for frailty factors and medications made little difference to effect estimates for individual comorbidities except for those for connective tissue disease (attenuated by medications) and dementia (attenuated by frailty factors, online supplementary file C).

#### Frailty factors

Visual problems was the only frailty factor associated with increased odds of hospitalisation in the final adjusted model, while the presence in the last year of bedsores, low weight/poor nutrition or incontinence had a negative effect on hospitalisation, as did residence in a nursing home (figure 1).

#### Medications/vaccinations

Patients with a prescription for antibiotics in the previous 8–28 days were less likely to be hospitalised after CAP than patients with no prescription in the previous 4 weeks, controlling for the other variables in the model. Oral steroid use was associated with increased odds of hospitalisation, but the strong effect of inhaled corticosteroids and other immunosuppressive medications disappeared after adjusting for comorbidities (see online supplementary file C). We did not observe a protective effect of statin use against hospitalisation. Influenza vaccination in the current influenza season lowered the odds of hospitalisation after CAP in the final adjusted model by 25% compared with those who had never been vaccinated. In contrast, receipt of pneumococcal vaccine showed no protective effect, with evidence of slightly increased odds among the group vaccinated ≥5 years ago (compared with unvaccinated).

#### Age/sex

In the final model, females remained at lower odds of hospitalisation than males, and hospitalisation odds increased with age up to 85–89 years. However, there was evidence that the effect of age on hospitalisation varied by sex (p_interaction_<0.001). In contrast to men, women aged ≥90 years were not at increased odds of hospitalisation compared with women aged 65–69 years after adjusting for comorbidities and other factors (table 2). The three-level model remained the most appropriate structure (compared with single or two-level) after the addition of all other factors to the model (all p<0.001).

**Table 2:** Results of the effect of age on post community-acquired pneumonia hospitalisation, in males and females

Age (years)	Male OR (95% CI)*	Female OR (95% CI)*
65–69	1	1
70–74	1.35 (1.13 to 1.61)	1.34 (1.10 to 1.61)
75–79	1.49 (1.26 to 1.76)	1.39 (1.16 to 1.66)
80–84	1.65 (1.40 to 1.96)	1.61 (1.35 to 1.93)
85–89	1.63 (1.36 to 1.94)	1.47 (1.23 to 1.75)
≥90	1.59 (1.31 to 1.94)	1.00 (0.84 to 1.19)

*Adjusted for: year, ischaemic heart disease, congestive heart failure, peripheral vascular disease, dementia, chronic lung disease, connective tissue disease, peptic ulcer, liver disease, diabetes, cancer, leukaemia/lymphoma, severe renal disease, cerebrovascular disease, neurological disease, disorders of the immune mechanism, terminal illness, recent carer, place of residence, vision problems, bed ulcer, underweight/nutritional replacement, incontinence/catheter, immunosuppressants (other than steroids), steroids, inhaled steroids, statins, antibiotics in previous 28 days, influenza vaccine.

### The effect of comorbidities on trends in post-CAP hospitalisations

After adjusting for all factors and for clustering, a marked increase in the percentage of CAP cases hospitalised over time remained, rising from 57% to 86% hospitalised over the study period. The wide range of comorbidities and other factors identified as risk factors for hospitalisation contributed very little to this increase (table 3).

**Table 3:** Average predicted probability (%) of hospitalisation within 28 days of community-acquired pneumonia diagnosis, by year

	Average predicted probability of hospitalisation (%)		
Year	No adjustment	Adjusted for age, sex and comorbidities*	Full model†
1998–2000	58	57	57
2001–2003	70	67	68
2004–2006	80	76	78
2007–2008	84	80	81
2009–2010	89	85	86

*Comorbidities: ischaemic heart disease, congestive heart failure, peripheral vascular disease, dementia, chronic lung disease, connective tissue disease, peptic ulcer, liver disease, diabetes, cancer, leukaemia/lymphoma, severe renal disease, cerebrovascular disease, neurological disease, disorders of the immune mechanism, terminal illness.

†As for comorbidities, with addition of: recent carer, place of residence, vision problems, bed ulcer, underweight/nutritional replacement, incontinence/catheter, immunosuppressants (other than steroids), steroids, inhaled steroids, statins, antibiotics in previous 28 days, influenza vaccine.

### Smoking

In total, 17 008 CAP events between 2007 and 2010 were included in the complete-case smoking analyses. After adjusting for age and sex, smokers had nearly three times the odds of being hospitalised than non-smokers (OR=2.83, 95% CI 2.25 to 3.56) with ex-smokers at nearly twice the odds (OR=1.88, 95% CI 1.59 to 2.23). After adjusting for comorbidities, smokers had 96% higher odds of hospitalisation than non-smokers, and ex-smokers 37% higher (see online supplementary file D).

### Further analyses

The age-adjusted and sex-adjusted odds of dying in the 28 days post-CAP decreased progressively over the study period, with patients in 2009–2010 having a 38% reduction in the odds of dying within 28 days of CAP compared with those in 2001–2003 (table 4). Length of hospital admission decreased slightly over the study period, from 8 (IQR 4–16) days in 1998–2000 to 7 (IQR 3–13) days in 2009–2010 (table 4). The percentage of short-term admissions (<2 days) increased over time from 11.7% to 14.1%. The majority of admissions (95.6%) occurred on the date of the CAP diagnosis.

**Table 4:** Post-CAP mortality, length of hospital admission and consultation behaviour on the day of CAP diagnosis, over time

	Year				
	1998–2000	2001–2003	2004–2006	2007–2008	2009–2010
OR for mortality within 28 days of CAP diagnosis*	1.01 (0.93 to 1.10)	1	0.84 (0.78 to 0.91)	0.73 (0.67 to 0.79)	0.62 (0.57 to 0.68)
Length of hospital admission median, (lower-upper quartile), days	8 (4–16)	8 (4–17)	8 (4–15)	7 (3–14)	7 (3–13)
0–2	468 (11.7)	660 (10.5)	981 (11.9)	999 (14.2)	1093 (14.1)
2–6	1138 (28.4)	1831 (29.2)	2595 (31.4)	2364 (33.6)	2731 (35.3)
7–13	1145 (28.6)	1737 (27.7)	2311 (27.9)	1827 (26)	1987 (25.7)
≥14	1256 (31.3)	2037 (32.5)	2383 (28.8)	1848 (26.3)	1930 (24.9)
Reason for admission, n (% of those hospitalised)					
Emergency: via A&E	2027 (50.6)	3760 (60)	5559 (67.2)	5073 (72.1)	5914 (76.4)
Emergency: via GP	1666 (41.6)	2016 (32.2)	2231 (27)	1522 (21.6)	1402 (18.1)
Emergency: via bed bureau	102 (2.5)	107 (1.7)	125 (1.5)	114 (1.6)	121 (1.6)
Emergency: via consultant outpatient clinic	26 (0.6)	42 (0.7)	43 (0.5)	41 (0.6)	42 (0.5)
Emergency: other means (including A&E from another place)	66 (1.6)	135 (2.2)	143 (1.7)	122 (1.7)	127 (1.6)
Transfer (non-emergency), elective, not known	120 (3)	205 (3.3)	169 (2)	166 (2.4)	135 (1.7)
Admitting diagnosis ICD10 Chapter X—diseases of the respiratory system	3798 (94.8)	5979 (95.4)	7939 (96)	6743 (95.8)	7426 (95.9)
Hospitalisations on CAP diagnosis date, n (% of those hospitalised)	3718 (92.8)	5893 (94.1)	7896 (95.5)	6804 (96.7)	7539 (97.4)
Relevant diagnosis on CAP date† (n, % all CAP)					
CPRD only	3234 (46.5)	3074 (34.3)	2546 (24.4)	1607 (19.1)	1265 (14.4)
HES only	2909 (41.8)	4644 (51.8)	5990 (57.4)	5266 (62.6)	5787 (65.7)
CPRD and HES	809 (11.6)	1249 (13.9)	1906 (18.3)	1538 (18.3)	1752 (19.9)

*Adjusted for age and sex using three-level model.

†General practice records included LRTI records as ‘potential CAP’, to allow for potentially conservative coding by GPs in the absence of radiographical confirmation of pneumonia (see Results section). HES records included any hospital admission record.

A&E, Accident and Emergency; CAP, community-acquired pneumonia; CPRD, Clinical Practice Research Datalink; GP, general practitioner; HES, Hospital Episode Statistics; ICD, International Classification of Diseases; LRTI, lower respiratory tract infection.

Emergency admissions recorded as being via Accident and Emergency (A&E) increased successively, from 50.6% of post-CAP admissions in 1998–2000 to 76.4% in 2009–2010. Conversely, emergency admissions coded as arriving via a GP fell from 41.6% to 18.1%, and there was a corresponding fall in the percentage of CAP events with a CAP or potential CAP record in the GP data, from 58% to 34%.

## Discussion

This is the first UK study to use large linked data sets to explore the factors associated with hospitalisation among CAP cases, and thus help identify high-risk patients for proactive case management. The factors we investigated had varying effects on hospitalisation. We were able to identify a wide range of patient factors that increased the odds of hospitalisation, including conditions common in older populations such as chronic lung disease, ischaemic heart disease, congestive heart failure, severe renal disease and diabetes. Analysis of the subset of data with near-complete recording of smoking status illustrated that smoking is also a strong risk factor for hospitalisation, independent of comorbidity status. Factors associated with decreased likelihood of hospitalisation included terminal illness, specific frailty factors and receipt of residential care. Individuals who had been recently vaccinated against influenza and those with recent antibiotic treatment were also less likely be hospitalised. An unexpected finding was that the oldest women (but not men) in our study were not at increased risk of hospitalisation compared with younger women in adjusted analyses; one possible explanation for this is a survivor effect among the oldest women.

Unlike previous studies that have reported pneumonia hospitalisation trends, we were able to demonstrate that hospitalisation after a CAP diagnosis is increasing independently of any trends in CAP incidence.^2^ ^18^ We additionally found that this increase does not appear to be driven by the underlying health and social issues of the older population. The average predicted probability of hospitalisation in the 28 days after CAP increased substantially in this population over the study period, from 57% to 86%, after extensive adjustment for changes in the prevalence of patient factors. All-cause mortality in the 28 days post-CAP and length of hospitalisation both decreased over the study period, with an increasing proportion of short-term (<2 day) admissions, suggesting that the increase in hospitalisation was not linked to increasing CAP severity. Owing to the lack of information on illness severity in these data, we cannot ascertain directly if less severely ill patients are being hospitalised over time, or whether hospital treatment has helped reduce mortality in the 28 days after admission.

We also found that over the study period progressively lower proportions of patients arrived in A&E after referral from their GP or with evidence that the GP had seen them for a LRTI on the day of CAP diagnosis, highlighting changes in patients’ health-seeking behaviour.

### Strengths

The use of large linked data sets meant that we could distinguish between community and hospital-acquired pneumonia and include non-hospitalised CAP episodes, which enabled assessment of risk factors specifically for hospitalisation. The very large linked data allowed thorough investigation of individual comorbidities and other variables, many of which are incompletely recorded or unrecorded in hospital admission data. The advantage of investigating individual comorbidities, compared with using a summary comorbidity score such as the Charlson score, is that we avoided masking of opposing associations of individual comorbidities on hospitalisation. For example, a Charlson score of 1 is given to a patient who has dementia, or to a patient with chronic lung disease. According to our analysis, a patient with dementia would have reduced odds of hospitalisation after CAP, whereas a patient with chronic lung disease would have increased odds. The linked data also allowed assessment of trends in hospitalisation independent of trends in CAP incidence, with detailed adjustment to account for any changes in the prevalence of patient risk factors for hospitalisation over time. The linked CPRD population is representative of the population of England, making our findings generalisable to the population at large, and the linked hospital data enabled us to identify the outcome (hospitalisation) with minimal misclassification. Further linkage to central mortality records allowed us to estimate mortality without restricting analyses to the subset of patients who died in hospital, thus avoiding changes in mortality over time due simply to changes in the relative proportions of patients who died in and outside hospital.

In contrast to previous studies that used only the first CAP episode in a year, we included patients with repeated episodes of CAP.^18^ ^19^ The association between specific comorbidities and hospitalisation could be particularly strong in this small but important subset of patients, and inclusion of their multiple episodes avoided potential underestimation of these associations.

### Limitations

Validity of recorded diagnoses is generally high in CPRD, and comorbidities and other risk factors that were only recorded after the hospitalisation were not included; thus, any misclassification of these factors is likely to be relatively small and non-differential with respect to the outcome.^20^ The linked data enriched our overall comorbidity coding, but smoking histories were under-recorded in the earlier years of the study period. The introduction of the Quality Outcomes Framework (QOF) in 2004 has improved the recording of smoking status in GP records, and analysis of the subset of data with near-full recording of smoking indicated its importance as a risk factor for hospitalisation after CAP. Similarly, while the use of GP data enabled investigation of some factors associated with frailty, these factors were not frequently recorded by GPs which limited our ability to assess fully their association with hospitalisation. Owing to the frailty indicators included in the data, we were unable to use an established measure of frailty such as the frailty phenotype or frailty index.^13^ ^14^ However, the frailty index includes several of the comorbidities we included individually in our model, such as diabetes, myocardial infarction and lung disease, and so use of this score may have led to overadjustment for these other important conditions. We examined a wide variety of variables; thus, estimates in the final model with a 95% CI close to including the null value should be interpreted with caution.

The HES pneumonia diagnoses used in this study have not been validated. There have been small localised reports of overdiagnosis of pneumonia in English hospitals, but trends over time at a national level have not been reported.^21–23^ As such we cannot exclude that overdiagnosis could have played a role in the increasing level of hospitalisation after CAP identified in this study. The forthcoming British Thoracic Society audit of CAP diagnoses will help to clarify this issue. Nevertheless, it is likely that the majority of these patients had a respiratory illness that was considered severe enough to merit hospitalisation, and these are of public health importance.

The data sources used in this analysis did not contain direct measures of pneumonia severity, such as those in the CURB score, and so severity of illness could not be measured directly.^24^ However, our aim was to establish patients’ pre-existing conditions which contributed to the increase in hospitalisation over time, not the mechanism by which this occurred (either by altering severity of CAP or by other means), and so we do not feel this detracts from our study findings. Furthermore, a recent systematic review highlighted suboptimal performance of CURB scores for oldest patients, and stressed the need to focus more on the presence of comorbidities and frailty in these patients.^25^ Similarly, due to the nature of the coding used in these data, we were not able to examine the severity of many of the comorbidities we investigated, and so could not directly assess whether increasing severity of these comorbidities over time could have explained some of the increase in hospitalisations during the study period. However, where we could distinguish categories of severity (eg, for diabetes, liver disease, renal disease and ischaemic heart disease), the likelihood of hospitalisation was very similar for those with severe and milder manifestations of the condition, and adjustment for these factors did not materially affect increasing hospitalisations.

### Findings in relation to other studies

We have previously shown that CAP incidence is rising among older individuals in the UK.^6^ This study confirms that hospitalisation following a CAP diagnosis is also increasing, with a growing percentage of cases hospitalised within 28 days of diagnosis. Our findings enhance those from previous studies that used stand-alone hospitalisation data, which have shown increasing hospitalisations for pneumonia without distinguishing increasing CAP incidence from an increasing tendency to hospitalise patients with CAP.^2^ ^18^ In particular, our analyses of hospitalisation trends update and extend those of a previous English study, which reported increasing pneumonia hospitalisation rates between 1997 and 2004 after less extensive adjustment for comorbidities, using the Charlson Index.^18^ Other studies that have investigated individual risk factors among older patients with CAP or LRTI have mostly been small and included fewer factors; some used hospitalisation or death as a composite outcome, which will obscure the opposing effects of conditions such as dementia or terminal care on these two outcomes.^19^ ^26–29^ Our finding that influenza vaccine receipt is associated with protection against hospitalisation after CAP is also consistent with the direction of effect shown in previous studies of influenza vaccine effectiveness against hospitalised CAP,^30^ ^31^ and studies showing a relative lack of long-term protection of pneumococcal vaccine, especially among those with underlying health conditions.^32^ ^33^ Our findings also add to those from a recent systematic review, which highlighted between-study heterogeneity in the association between statin use and outcomes of pneumonia.^34^ The reduction in mortality seen over the study period echoes that from the earlier English study which focused on in-hospital mortality, as well as CAP mortality studies from Europe and the USA.^18^ ^35^ ^36^

### Meaning, explanations and implications for future research

The risk factors identified in this study will be of benefit to clinicians managing patients in primary care settings, by helping to identify patients at high risk of unplanned admission to hospital who are in need of proactive case management. Our findings will further inform discussions with these patients about protecting against infection risk and seeking early treatment for symptoms.

Frailty is currently a health priority in the UK. The requirement in the 2014 general practice contract for increased identification of vulnerable older members of the practice population may result in better recording of frailty in general practice data and enable more thorough investigation of its effects on hospitalisation in future research.^37^ The latter will be helped by a new primary care electronic Frailty Index, currently under development in England.^38^

Despite their importance in identifying high-risk patients, our adjusted analyses show that increasing prevalence of comorbidities and frailty are not driving the increase in hospitalisation rates. Declining mortality and length of hospital stay indicate that this is not due to increasing disease severity. What then explains the increasing hospitalisation trend? The guidelines for management of CAP issued by the British Thoracic Society have not changed significantly over the study period.^39^ ^40^ However, the diagnostic accuracy of pneumonia may have changed over time. An emergency department-based US study found that the accuracy of pneumonia diagnoses decreased after the change of a core quality measure (time to first antibiotic dose) from 8 to 4 h.^41^ In England, the introduction of the 4 h A&E waiting time target in 2004 could have had a similar effect.

In addition, changes to service provision and utilisation have been highlighted as playing a role in the increase, with the change in out-of-hours access to GPs during the study period.^42^ The effect of this is difficult to measure directly, but we found decreasing emergency admissions over time arriving via a GP, and a decreasing proportion of patients with a CAP or potential CAP recorded by their general practice on their CAP diagnosis date. Studies have also shown that the increase in emergency admissions among older individuals in England is not restricted to pneumonia but are seen for a range of other conditions, and that the percentage of patients who attended A&E and were then admitted rose by over a third between 2003 and 2012, with 75% of this rise attributed to increasing emergency admissions and 25% to an increase in A&E attendance.^43^ Thus, an increasing tendency to hospitalise, coupled with an increasing inclination of patients to present to A&E rather than to their general practice, may be a main driver of the growth in hospitalisation after CAP. It would be interesting to compare our results with those from equally detailed studies that use linked data to investigate risk factors and hospitalisation trends for other conditions, such as COPD or cellulitis. Results from these studies would allow further interpretation of whether increasing hospitalisation and decreasing primary care consultation trends are not specific to CAP among older adults.

Our study, based on very large numbers (minimising random error) and with the ability to adjust hospitalisation rates for many factors, supports the argument that focusing on high-risk patients, while important for risk stratification, will not appreciably reduce emergency admissions.^42^ If the incidence of CAP among those aged ≥65 years also continues to increase, these combined trends will place a joint expanding burden on the health service.
